# Introducing a Virtual Fracture Clinic Increases Efficiency and Reduces Costs in Torus Fracture Management

**DOI:** 10.1097/pq9.0000000000000202

**Published:** 2019-11-25

**Authors:** Sheena Seewoonarain, Satish Babu, Dhrumin Sangoi, Adhish Avasthi, David Ricketts

**Affiliations:** From the * East Kent University NHS Foundation Trust; † Frimley Health NHS Foundation Trust; ‡ Western Sussex NHS Health Trust; § Brighton and Sussex Univeristy Hospitals NHS Trust

## Abstract

Torus fractures of the distal radius are both a common pediatric forearm fracture and a common source of referral to orthopedic departments. Management of these injuries has moved away from traditional casting methods to using splints and softcasts.

**Aim:**

With the recent introduction of a virtual fracture clinic (VFC), we sought to improve our compliance with guidelines while ensuring all patients undergo review in the clinic.

**Methods:**

We audited the management of patients before and after the introduction of the VFC with new management protocols for torus fracture patients.

**Results:**

There was a 51% decrease in patients managed using plaster of Paris with 5% treated with a softcast and 59% using a splint.

**Outcome:**

Using the VFC can improve the management of patients with torus fractures and provide a cost-saving and a more positive experience for patients.

## INTRODUCTION

Torus or buckle fractures of the distal radius are the most common pediatric forearm fracture and are a common source of referral to orthopedic departments. They occur in the transition zone between metaphyseal and diaphyseal bone.^1^

Historically torus fractures were managed with cast immobilization, and serial radiographic follow-up in an orthopedic outpatient setting.^2^ The recent National Institute for Health and Care Excellence guidelines advocate management of torus fractures with nonrigid casts or splints. The patient can remove both of these without the need for further radiographs or follow-up.^3^

Virtual fracture clinics (VFCs) have become increasingly popular. They allow compliance with British Orthopaedic Association Standards for Trauma Guidelines. These require that all patients with an acute orthopedic injury should be reviewed within 72 hours in a new fracture clinic. The use of VFCs is particularly effective for patients requiring nonoperative management with home management protocols.^4^

This study aimed to assess the efficiency gains (of both fracture management and costs) resulting from the management of torus fractures by a VFC.

## MATERIAL AND METHODS

Location of study: We performed this study at a district general hospital: St Richard’s Hospital, Chichester, United Kingdom.

Study design: The study took the format of a closed-loop audit with retrospective data collection.

Data collected: We collected data, including patient details, radiographs, follow-up, and management plans over 2 six-month periods.

Inclusion criteria: We included all patients under the age of 16 years who presented to the emergency department with radiographic confirmation of a torus fracture of the distal radius. Patient notes, radiographs, clinic letters, and appointments were used to determine subsequent management.

Exclusion criteria: Patients 16 years of age and above, greenstick fractures and those with associated ulnar styloid fractures.

First audit cycle: The first cycle of data collection took place between November 1, 2015 and April 30, 2016 inclusive. This audit was before the introduction of the VFC in October 2016.

A mixture of casualty doctors, emergency nurse practitioners, and orthopedic on-call doctors reviewed patients. Patients were managed with a cast or splint and referred to the fracture clinic at the next available appropriate appointment.

Second audit cycle: The second cycle of data collection took place after the introduction of the VFC between November 1 and April 30, 2017.

**Fig. 1.**
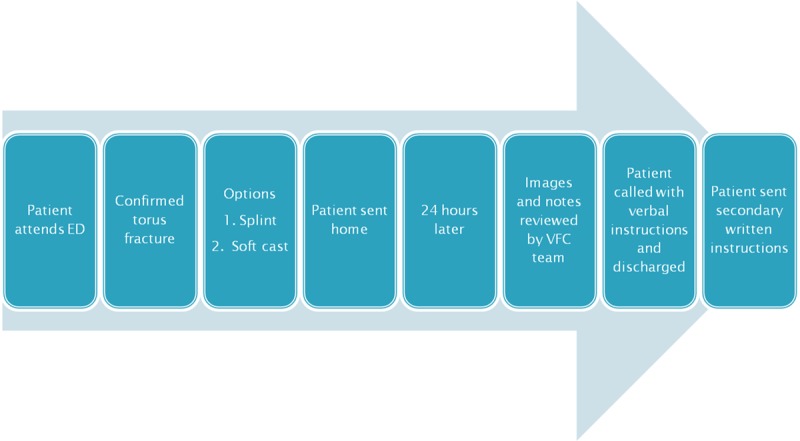
Flowchart of VFC protocol for patients with torus fractures. ED, Emergency Department.

After we introduced the VFC (staffed by a dedicated physiotherapy team) in November 2017, patients attending the emergency department were given a splint or softcast by the emergency department with verbal and written instruction protocols supplied by the VFC. These patients bypassed the on-call orthopedic team. Their injuries were reviewed in the VFC during the next working day by the on-call orthopedic consultant. The on-call orthopedic consultant assessed the patient’s radiographs and documentation from the accident and emergency department. All patients were called the same day to discuss the advice given by the consultant and sent secondary documented advice (Fig. 1). There was no VFC on the weekends. The reviews on a Monday morning were of referrals from the previous 72 hours.

There were 2 possible outcomes from the VFC review. The first was discharge following a phone call discussion regarding management with this also emailed or posted to the patient. The second was a review in the fracture clinic.

## RESULTS

### Fracture Management

Pre-VFC cycle: this cycle included 39 patients. Of these, 34/39 (87%) were treated in plaster of Paris and 5/39 (13%) with a splint. All 39 patients were assessed in a fracture clinic.

**Fig. 2.**
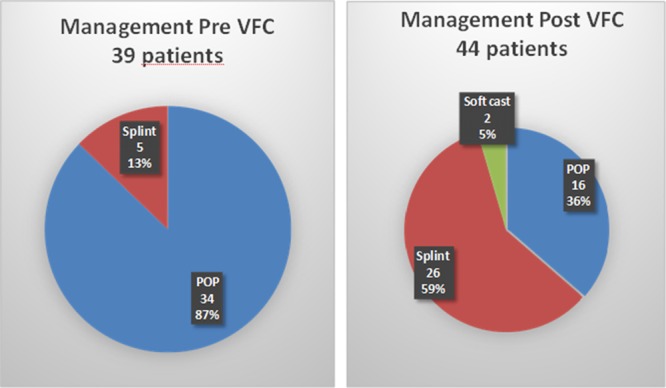
Comparison of treatment of pediatric patients before and after the VFC. POP, plaster of Paris.

Post-VFC cycle: this cycle included 44 patients. Of these 16/44 (36%) were treated with a cast, 2/44 (5%) in a softcast and 26/44 (59%) using a splint (Fig. 2).

Table 1 demonstrates the parameters of discharge, repeat radiographs, and time in plaster of Paris before and after the introduction of the VFC.

**Table 1.**
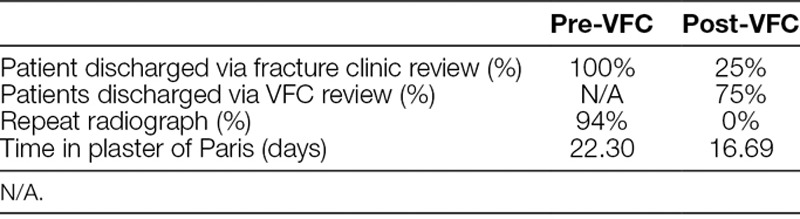
Comparison of discharge from care method, repeat radiograph and time in plaster between the pre-VFC cycle and post-VFC cycle

### Costs

We compared the costs of torus fracture management before and after the introduction of the VFC. Our trust costs the traditional fracture clinic follow-up an appointment at £154. A VFC review is £74. £1 is equal to $1.75 at the time of the study.

The price of a splint is £4. Any grade of nurse or doctor can apply the splint. The estimated cost of the required materials for plaster of Paris is £3.50. The added cost of trained personnel, for example, plaster technician, to fit and remove the plaster of Paris is £7.50 leading to a total cost of £11. A soft cast was estimated at £12.90 for materials and £3.75 for the plaster technician time so a total of £16.65. A full cast costs £4.86 for materials and £5.82 of plaster technician time so a total of £10.68. A comparison was made of these costs pre- and post-VFC clinic (Table 2).

**Table 2.**
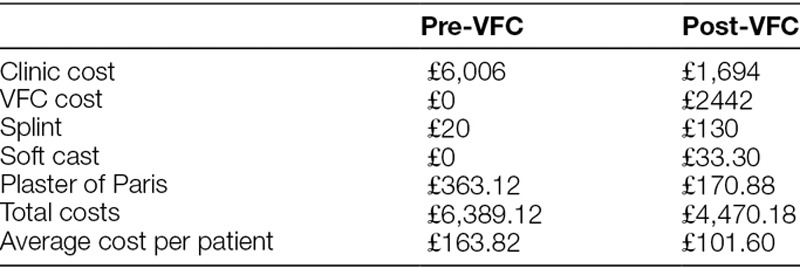
Comparison of costs pre- and post-VFC with the total costs and average cost per patient

## DISCUSSION

We believe that VFC has many advantages in the management of torus fractures. Primarily, we found that the VFC enabled us to meet National Institute for Health and Care Excellence guidelines regarding follow-up and improve fracture management. All patients were able to have a VFC review within 72 hours of presentation. The VFC discharged 75% of patients following a VFC review.

Second, we were able to provide a cost-saving by using the VFC to be £61.22 per patient based on the cohort of patients in this study. We also noted a reduction in exposure to radiation. By encouraging discharge through the VFC allowed a 94% reduction in repeat radiographs so that no patients were exposed to avoidable radiation in the post VFC cycle.

In addition to the above benefits, we believe that the VFC service provides a decrease in inconvenience for children and their families: Parents were able to remove the cast or splint at home without the need for travel to hospital for an outpatient appointment. This benefit avoided waiting times, logistical issues in attending the appointment, and taking time off from work and school.^10^ Morris and Bell reported that every clinic attendance cost 0.25 workdays, 0.18 days wages, and 0.54 days of schooling.^8^

The move to VFC is part of a wider shift away from conventional fracture clinic reviews. Protocol-driven VFC management reduces variation in practice and avoidable clinic appointments.^10,11^ It also avoids poor patient experiences in outpatients due to delays and cancellations. The Audit Commission estimated that of all orthopedic outpatient appointments in the United Kingdom in 2013, 25% were delayed by 30 minutes, 4 million were canceled, and for 6.9 million appointments patients did not arrive.^10,11^ Huntley^12^ reported that half of the pediatric referral to fracture clinic were avoidable, and 15% of attendances were inappropriate.

Thirty-six percent of patients were managed with a cast indicating that there was still space for improvement within our practice. There were likely several reasons for this: the VFC was introduced a month before the second cycle of the audit. Staff were not used to the protocols introduced. Protocol posters are now available in the emergency department. Our current VFC protocol bypasses the need for emergency doctors to refer these patients to the on-call orthopedic team.

One limitation of our study is that patients were not followed up with regards to patient outcomes and potential further surgery in the first cohort of patients. There were no adverse outcomes reported in the second cohort of patients that were managed using the VFC. Patients were also able to contact the VFC if they had any further concerns.

## CONCLUSIONS

We recommend the use of VFC for the management of torus fractures. This approach leads to improved efficiency and cost savings while meeting national standards.

## DISCLOSURE

The authors have no financial interest to declare in relation to the content of this article.
